# Aerobically trained older adults show impaired resting, but preserved exercise-induced circulating progenitor cell count, which was not improved by sprint interval training

**DOI:** 10.1007/s00424-022-02785-6

**Published:** 2023-02-14

**Authors:** Zerbu Yasar, Mark D. Ross, Christopher J. Gaffney, Ruth D. Postlethwaite, Russell Wilson, Lawrence D. Hayes

**Affiliations:** 1 Active Ageing Research Group, Institute of Health, University of Cumbria, Lancaster, UK; 2 School of Applied Sciences, Edinburgh Napier University, Edinburgh, UK; 3 School of Energy, Geoscience, Infrastructure and Society, Heriot-Watt University, Edinburgh, UK; 4 Lancaster Medical School, Faculty of Health and Medicine, Lancaster University, Lancaster, UK; 5 Faculty of Health and Life Sciences, Coventry University, Coventry, UK; 6 Sport and Physical Activity Research Institute, School of Health and Life Sciences, University of the West of Scotland, Glasgow, UK

**Keywords:** Ageing, Endothelial, Endothelial progenitor cells, HIIT, Sprint, Vascular

## Abstract

Older adults exhibit a reduced number and function of CD34 + circulating progenitor cells (CPC), a known risk factor for cardiovascular disease. Exercise promotes the mobilisation of CPCs from bone marrow, so whether ageing per se or physical inactivity in older age reduces CPCs is unknown. Thus, this study examined the effect of age on resting and exercise-induced changes in CPCs in aerobically trained adults and the effect of 8 weeks of sprint interval training (SIT) on resting and exercise-induced CPCs in older adults. Twelve young (22–34 years) and nine older (63–70 years) adults participated in the study. Blood was sampled pre and immediately post a graded exercise test to exhaustion in both groups. Older participants repeated the process after 8 weeks of SIT (3 × 20 s ‘all-out’ sprints, 2 × a week). Total CPCs (CD34^+^) and endothelial progenitor cells (EPCs: CD34^+^KDR^+^) were determined by flow cytometry. Older adults exhibited lower basal total CD34^+^ CPCs (828 ± 314 vs. 1186 ± 272 cells·mL^−1^, *p* = 0.0149) and CD34^+^KDR^+^ EPCs (177 ± 128 vs. 335 ± 92 cells·mL^−1^, *p* = 0.007) than younger adults. The maximal exercise test increased CPCs in young (CD34^+^: *p* = 0.004; CD34^+^KDR^+^: *p* = 0.017) and older adults (CD34^+^: *p* < 0.001; CD34^+^KDR^+^: *p* = 0.008), without difference between groups (*p* = 0.211). SIT did not alter resting or exercise-induced changes in CPCs in the older cohort (*p* > 0.232). This study suggests age per se does not impair exercise-induced CPC counts, but does lower resting CPC counts.

**Supplementary Information:**

The online version contains supplementary material available at 10.1007/s00424-022-02785-6.

## Introduction

Advancing age is associated with an increased risk of onset and progression of cardiovascular disease (CVD) [29], often attributed to comorbidities such as hypertension [44, 56], diabetes and hyperlipidemia [56]. Advancing age is also associated with a reduced endothelial function [3] and vascular reparative capacity, indicated by reduced circulating progenitor cell (CPC) count and function [15, 55, 61]. These CPCs, defined as CD34^+^ progenitors, offer regenerative benefits to the vascular endothelium by taking part in endothelial repair by paracrine means [27]. Studies have demonstrated that individuals with a lower resting number of these cells are at a greater risk of cardiovascular and all-cause mortality [40, 42], therefore, increasing CPC number and function may be of clinical significance.

Exercise acutely mobilises CPCs into the peripheral blood compartment in the recovery period post-exercise [52, 63] and is intensity- and duration-dependent [28]. This is thought to be due to mobilisation from bone marrow, promoting CPC migration from the bone marrow niche, and into circulation, where these cells exert their vaso-reparative function. Interestingly, the extent to which CPCs are increased in response to an exercise stressor is associated with future cardiovascular (CV) risk in CVD patients [37], with a blunted response associated with an increased risk of adverse events. Previous work has also demonstrated older adults display attenuated CPC response to submaximal exercise compared to younger individuals [45],therefore, interventions may be required to promote not only the resting number of CPCs but also the exercise responsiveness, which may be related to bone marrow resident number, and capillarity of the bone marrow to allow for more CPCs to enter the circulation.

Sprint interval training (SIT) is a novel, time-efficient mode of exercise which is known to promote markers of cardiometabolic health, such as aerobic capacity [8, 24, 65, leanness [14, 38], and lowered fasting blood glucose [1]. One study has shown that SIT in young, healthy women was effective at increasing CD34^+^ CPC resting number but not function [12]. Therefore, SIT may be an effective intervention for promoting changes in CPC counts in older adults who demonstrate lower resting numbers, which may subsequently improve vascular repair capacity and reduce future CVD risk. However, one difficulty in discerning the effect of age specifically on CPCs (or any physiological parameter) is the age-associated reduction in physical activity [36, 54, 58]. As such, it is important to differentiate the effect of age, rather than age, *in addition* to years of reduced physical activity on physiological parameters, and in this case CPCs. In this context, we believe it is important to match participants for fitness or physical activity to truly examine the effect of age on CPCs.

The present investigation aimed to examine the effect of age on resting and exercise-induced changes in CPCs in aerobically trained young and older individuals. A secondary aim was to examine the effect of novel SIT stimuli in the older group on CPCs (both basal and exercise-induced changes). It was hypothesised a priori that older adults would display a lower number of resting CPCs, an attenuated CPC rise in response to a maximal exercise stressor, and that an 8-week SIT protocol would recover resting and exercise-induced changes in CPCs to that similar of the younger cohort.

## Materials and methods

### Participants

**Fig. 1:**
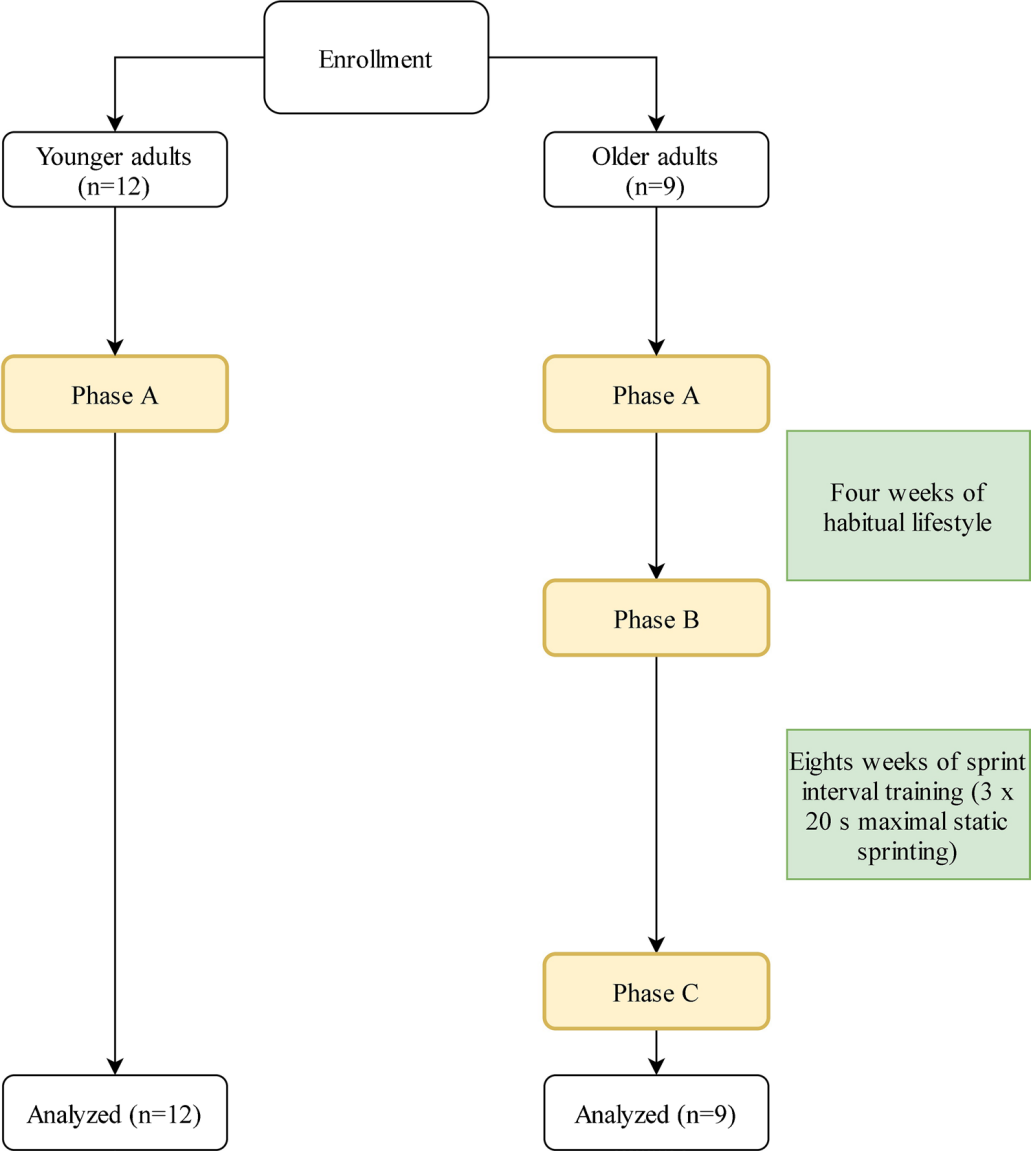
Schematic representation of the methodological flow. PPO = peak power output. $\dot{{\varvec{V}}}$ O_2max_ = maximal oxygen uptake

Two cohorts were recruited for this study, younger (*n* = 12, 28 ± 5 years of age, body mass index [BMI]: 24.5 ± 2.2 kg·m^2^) and older (*n* = 9, 67 ± 3 years of age, BMI: 22.5 ± 2.0 kg·m^2^) adults, who regularly participated in a weekly minimum of 150 min∙wk^−1^ of moderate or high-intensity exercise for at least 6 months prior to participating in the study and continued habitual physical activity for the duration of the study. The older females in the study were post-menopausal. Participants were free of exercise-contraindicating disease (metabolic, cardiovascular and renal) or injury as determined by a Physical Activity Readiness Questionnaire (PAR-Q) and American College of Sports Medicine (ACSM) pre-exercise participation screening, without any requests for medical clearance submitted within the cohort [43]. This study was carried out in accordance with the Declaration of Helsinki and approved by the University of Cumbria Research Ethics Committee. Written informed consent was obtained from all participants prior to study commencement, and subjects were excluded if they presented with atrial fibrillation. Descriptive statistics for participants are shown in Table 1 and further described in the results section. Participants attended all sessions with exercise-suitable clothing and footwear. The younger cohort attended a single test session, whilst the older cohort attended two separate testing sessions,before (pre) and five days after the final training session of the 8-week SIT intervention (post) (Fig. 1). Participants were fasted overnight before all testing sessions, breaking their fast only after the testing session. As this study was a secondary analysis (primary outcome: muscle power), no a priori power calculation was performed specifically for CPCs.

**Table 1:** Participant characteristics with *t*-test alpha values for baseline comparisons between young and older participants and pre- to post-sprint interval training comparisons

	Young	Older pre-SIT	Older post-SIT	Young vs older pre-SIT	Older pre-SIT vs post-SIT
Age (years)	28 ± 5	67 ± 3			
Sex (% female)	8%	22%			
Height (cm)	179 ± 7	174 ± 11			
Body mass (kg)	78.5 ± 7.6	68.3 ± 10.4	67.9 ± 9.1	*p* = 0.008	*p* = 0.082
BMI (kg·m^2^)	24.4 ± 2.2	22.5 ± 2.0	22.4 ± 1.7	*p* = 0.052	*p* = 0.107
Systolic blood pressure (mmHg)	123 ± 7	125 ± 13	125 ± 16	*p* = 0.299	*p* = 0.465
Diastolic blood pressure (mmHg)	72 ± 7	73 ± 6	72 ± 6	*p* = 0.286	*p* = 0.085
Mean arterial pressure (mmHg)	89 ± 6	91 ± 7	89 ± 7	*p* = 0.246	*p* = 0.299
$\dot{V}$ O_2max_ (ml·kg·min^−1^)	51.6 ± 12.6	37.4 ± 7.6	39.5 ± 8.7	*p* = 0.003	*p* = 0.113

Values shown are mean ± SD

*BMI*, body mass index; $\dot{\mathrm{V}}$ O_2max_, maximum oxygen uptake

### Blood draws and analysis

Participants arrived at the exercise physiology laboratory between 08.00 and 11.00 h, following an overnight fast and having abstained from strenuous physical activity for a minimum of 48 h. Participants were reminded to maintain standardised conditions prior to each assessment point which included arriving in a hydrated state and having abstained from caffeine and alcohol consumption for 24 h. Following 20-min supine rest, blood was sampled from the antecubital vein using the standard venepuncture method into sterile TransFix® K3EDTA vacutainer tubes (TransFix, Cytomark Ltd, UK). These tubes contained Tranfix® solution which preserved cell antigens on mononuclear cell subsets for delayed flow cytometric analysis. The use of this preserving solution for progenitor cell analysis has been validated for flow cytometric analysis of samples for up to 7 days post-collection [20]. Blood samples were collected at the same time of day for each participant to control for biological variation and minimise within-participant variation [13]. Resting blood draws were completed prior to any exercise testing.

### Anthropometry

Height was measured to the nearest 0.1 cm and mass to the nearest 0.01 kg using a Seca 286 measuring station (Birmingham, UK), from which body mass index (BMI) was derived by dividing mass by the square of height (kg·m^2^).

### Peak power output

Peak power output (PPO) was established using the 6-s Herbert test [16] on an air-braked cycle ergometer (Wattbike Ltd., Nottingham, UK), which consisted of a maximal 6-s sprint from a standing start.

### Maximal oxygen uptake ($\dot{{\varvec{V}}}$ O2max) 

At least five min after PPO determination, $\dot{V}$ O_2max_ was determined using a Cortex II Metalyser 3B-R2 (Cortex, Biophysik, Leipzig, Germany). Expiratory airflow was achieved using a volume transducer (Triple V® turbine, digital) connected to an oxygen (O_2_) analyser. Expired gases were analysed for O_2_ with electrochemical cells and for carbon dioxide (CO_2_) output with an infrared analyser. The metalyser was calibrated according to the manufacturer’s guidelines prior to each test. After a 60-min warm-up period, the O_2_ and CO_2_ sensors were calibrated against environmental air in addition to reference gas of known composition (5% CO_2_, 15% O_2_ and 80% N_2_) with volume calibrated by five inspiratory and expiratory strokes using 3-L pump. Prior to the determination of $\dot{V}$ O_2max_, a chest strap heart rate monitor was attached to participants’ chests, with heart rate measured continuously throughout the test (Polar F1, Polar, Finland). The cycle ergometer (Wattbike Pro, Wattbike, UK) was adjusted to the manufacturer’s guidance. The saddle height was adjusted relative to the crank position, and the foot was secured to a pedal with straps with the participants’ knee at almost full extension (~ 170°). Participants mounted the cycle ergometer, and a rubber face mask was fitted (Hans Rudolph Inc, USA), which was attached to the Cortex II Metalyser 3B-R2. $\dot{V}$ O_2_ and $\dot{V}$ CO_2_ were recorded continuously throughout the test.

Prior to the graded exercise test to exhaustion, participants completed a 3-min warm-up at an intensity equivalent to ~ 10% of PPO. Subsequently, participants cycled at the increasing intensity with 25 W∙min^−1^ increments until they reached volitional exhaustion, with a rating of perceived exertion (RPE; 0–10 scale) [8] recorded in the last 10 s of each stage. Immediately following volitional exhaustion, participants had their index finger cleaned using a disinfectant wipe, and then a lancet lacerated a fingertip to obtain a blood sample for to measure blood lactate concentration [BLa] (Lactate Pro 2, Arkray, Japan). $\dot{V}$ O_2max_ was confirmed when participants achieved a minimum of any four of the following criteria: $\dot{V}$ O_2_ plateau, RER ≥ 1.10, peak heart rate within 10 beats of age-predicted maximum, [BLa] ≥ 8 mmol·L^−1^, and final RPE of ≥ 9.

### Flow cytometry

Flow cytometric analyses were performed on Tranfix® (Cytomark Ltd, UK) within 7 days post-blood sample collection. Briefly, 100 µL of whole blood was incubated with fluorescent antibodies against known cell surface antigens for determining CPCs. These included anti-CD34 BV650, anti-CD45 BV786 and anti-KDR PE (BD Biosciences, USA). 7-AAD (BD Biosciences, USA) staining was also performed to remove non-viable cells from analysis. After 45-min incubation, erythrocyte lysis was performed using lysis buffer (BD FACS™ Lysing Solution, BD Biosciences, USA). Quantification of CPC counts was then performed on a 12-colour flow cytometer (BD FACS Celesta, BD Biosciences, USA). 500,000 CD45^+^ events were collected for each sample to ensure sufficient data for rare cell populations. After gating for CD45^+^ events, non-viable 7AAD^+^ events were excluded, with subsequent gating for CD34^+^ events and lastly for KDR^+^ events. Appropriate negative tubes were used to determine positive and negative events for each targeted antibody. Percentage events were collected as % mononuclear events, in addition to the calculation of cells·mL^−1^ using dual platform analysis. To do so, lymphocytes were enumerated using differential haematology analysis (XS1000i, Sysmex, UK) and % of lymphocyte events were used with lymphocyte number to determine CPCs as cells·mL^−1^. Analyses of flow cytometric data were performed using BD FACSDiva™ software (BD Biosciences, USA). Gating parameters can be found in Supplementary Fig. 1.

For pre- to post-exercise comparisons, changes in blood volume due to hemoconcentration were accounted for using measured haematocrit and haemoglobin obtained from automated haematology analysis using equations by Dill and Costill [7].

### Exercise training

**Fig. 2:**
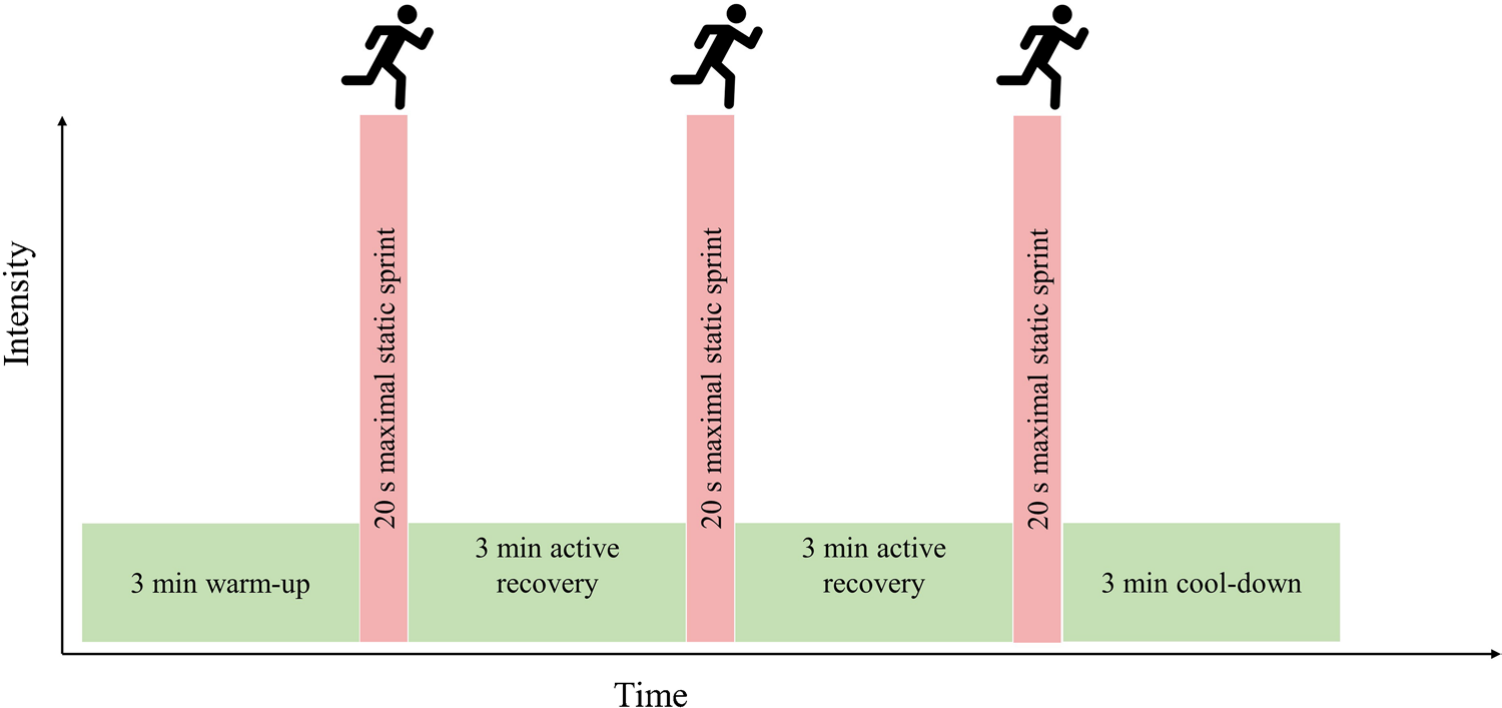
Schematic representation of the sprint interval session. Participants performed this session twice weekly for eight weeks

In the present study, the older adults underwent an 8-week SIT intervention involving 3 × 20 s ‘all-out’ sprints twice per week. The two SIT sessions per week were ≥ 72 h apart, as our pilot work suggested older adults would be suitably recovered from SIT in this timeframe [62]. Participants avoided strenuous physical activity 24 h prior to SIT sessions whilst maintaining habitual physical activity according to self-reporting. Participants warmed up for a period of 3 min at a self-paced intensity by performing static running. Participants then performed three 20-s static sprints at an ‘all-out’ intensity, interspersed by 3-min self-paced recovery phases. Following the final sprint, a 3-min self-paced cool down was performed (Fig. 2). During all sprints, participants were instructed to raise their feet to approximately knee height, with loud verbal encouragement throughout each sprint. No dietary guidance or monitoring was provided during the training, except for the fasted testing sessions.

### Statistical analysis

All data were assessed for normal distribution using the Kolmogorov–Smirnov test for normality. All data were normally distributed. To assess the differences in resting and exercise-induced changes in CD34^+^ and CD34^+^KDR^+^ CPCs between young and older adults, 2 × 2 mixed factorial analyses of variance (ANOVA) were performed with Tukey’s multiple comparisons post-hoc tests performed where necessary. Resting CPC counts were compared as both % MNCs and as cell·mL^−1^, whereas exercise-induced changes (pre- to post-exercise) were compared for the main effects of exercise, age and intervention were compared as cells $\cdot$ mL^−1^. The delta (Δ) change in cells·mL^−1^ was compared between young and older adults by means of an independent *t*-test. To compare the effect of the SIT intervention in older adults, a mixed effects model was performed to compare resting and exercise-induced CPCs between pre- and post-SIT and between these data with the young cohort. Data were analysed using GraphPad Prism (GraphPad Prism 9.1.0, GraphPad Software Inc, USA). Data are presented as mean ± SD (95% confidence intervals [CI]) without subjective terminology, and alpha levels are reported as exact *P* values, without dichotomous interpretation of ‘significant’ or ‘non-significant’ as advised by the American Statistical Association [21]. Effect sizes are reported using Cohen’s *d* (difference in means ÷ pooled standard deviation [SD]) and interpreted using guidelines for gerontology [5], which are *d* ≥ 0.15 = small, *d* ≥ 0.40 = moderate, and *d* ≥ 0.75 = large.

## Results

### Influence of age on resting and exercise-induced changes in CPC counts

**Fig. 3:**
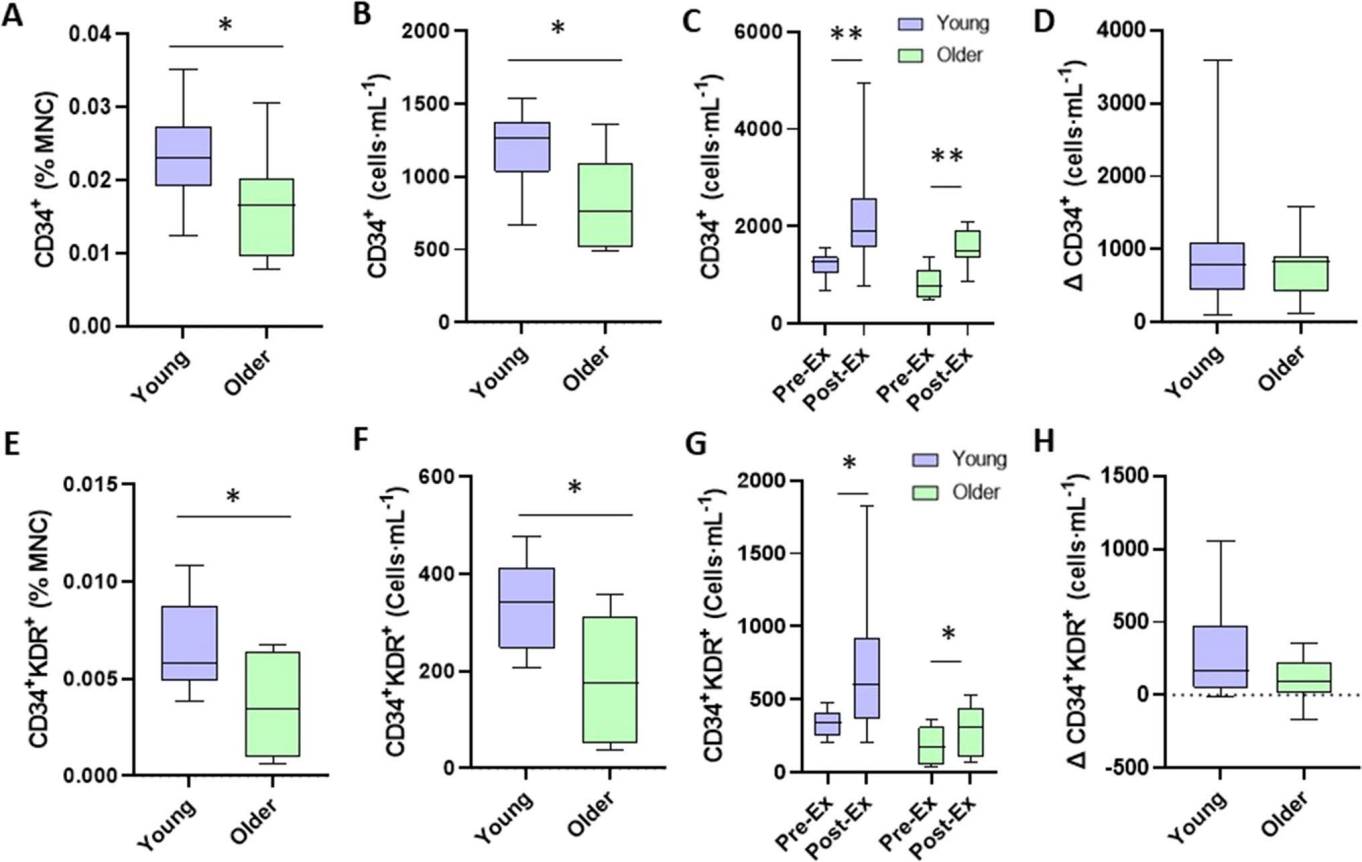
CD34^+^ and CD34^+^KDR^+^ CPC resting and exercise-induced changes in CPC counts in young (*n* = 12) and older (*n* = 9) trained adults. **A**, **B** and **E**, **F** display differences between young and older adults in CD34^+^ (A: % MNC, B: cells·mL^−1^), CD34^+^KDR^+^ (E: % MNC, F: cells·mL^−1^). **C**, **D** and **G**, **H** display changes in CPCs from pre- to post-exercise between young and older adults (**C**: CD34^+^ changes from pre- to post-exercise; **D**: Δ change in CD34^+^ CPCs; **G**: CD34^+^KDR^+^ changes from pre- to post-exercise, **H**: Δ change in CD34^+^KDR.^+^ CPCs). *p < 0.05, **p < 0.005

Older and younger adults circulating the number of CD34^+^ CPCs as a percentage of MNCs were 0.0159 ± 0.0073% [0.0103–0.0216% 95% CI] and 0.0233 ± 0.0060% [0.0195–0.0271% 95% CI], respectively (old vs young; *p* = 0.026, *d* = 1.10). Older and younger adults circulating the number of CD34^+^ CPCs were 828 ± 314 [587–1070 95% CI] cells·mL^−1^ and 1186 ± 272 [1012–1359 95% CI] cells·mL^−1^, respectively (old vs young; *p* = 0.015 *d* = 1.22). Older and younger adults circulating CD34^+^KDR^+^ EPCs as a percentage of MNCs were 0.0034 ± 0.026% [0.0014–0.0054% 95% CI] and 0.067 ± 0.023% [0.0052–0.0082% 95% CI] of MNCs, respectively (old vs young; *p* = 0.008, *d* = 2.59). Older and younger adults’ number of circulating CD34^+^KDR^+^ EPCs were 177 ± 128 [79–275 95% CI] cells·mL^−1^ and 335 ± 92 [227–394 95% CI] cells·mL^−1^ respectively (old vs young; *p* = 0.007 *d* = 1.42; Fig. 3).

The number of CD34^+^ progenitor cells in older adults before and after the graded exercise test to exhaustion were 828 ± 314 [587–1070 95% CI] cells·mL^−1^ and 1582 ± 381 [1290–1878 95% CI] cells·mL^−1^, respectively (pre- to post-exercise; *p* < 0.001, *d* = 2.16). The number of CD34^+^ progenitor cells in the young adults before and after the graded exercise test to exhaustion were 1186 ± 272 [1012–1359 95% CI] cells·mL^−1^ and 2134 ± 1049 [1467–2800 95% CI] cells·mL^−1^, respectively (pre- to post-exercise; *p* = 0.004, *d* = 1.23). CD34^+^KDR^+^ EPCs in the older adults before and after the graded exercise test to exhaustion were 177 ± 128 [79–275 95% CI] cells·mL^−1^ and 280 ± 176 [145–416 95% CI] cells·mL^−1^, respectively (pre- to post-exercise; *p* = 0.008, *d* = 0.67). CD34^+^KDR^+^ EPCs in the young adults before and after the graded exercise test to exhaustion were 225 ± 92 [277–394 95% CI] cells·mL^−1^ and 717 ± 493 [403–1030 95% CI] cells·mL^−1^. respectively (pre- to post-exercise; *p* = 0.017, *d* = 1.39). The older cohort’s ∆CD34^+^ from pre- to post-graded exercise test to exhaustion was 754 ± 430 [424–1084 95% CI] cells·mL^−1^, whilst the young cohort’s ∆CD34^+^ from pre- to post-graded exercise test to exhaustion was 948 ± 907 [372–1524 95% CI] cells·mL^−1^ (old vs young; *p* = 0.775, *d* = 0.27). The older cohort’s ∆CD34^+^KDR^+^ CPCs from pre- to post-graded exercise test to exhaustion was 103 ± 157 [− 18–224 95% CI] cells·mL^−1^, whilst the young cohort’s ∆ CD34^+^KDR^+^ CPCs from pre- to post-graded exercise test to exhaustion was 299 ± 365 [66–531 95% CI] cells·mL^−1^ (old vs young; *p* = 0.212, *d* = 0.70).

### Effect of 8 weeks of sprint interval training on resting and exercise-induced CPC changes in trained older adults

In terms of basal concentrations in the older group, CD34^+^ CPC as a percentage of MNCs was 0.0159 ± 0.0073% MNC [0.0103–0.0216% 95% CI] and 0.0148 ± 0.0055% MNC [0.0106–0.0190% 95% CI] pre- and post-training, respectively (*p* = 0.694, *d* = 0.17). CD34^+^ CPC in cells·mL^−1^ was 828 ± 314 [587–1070 cells·mL^−1^ 95% CI] and 765 ± 299 cells·mL^−1^ [535–995 cells·mL^−1^ 95% CI] at pre- and post-training, respectively (*p* = 0.602, *d* = 0.20). CD34^+^KDR^+^ CPC as a percentage of MNCs pre- and post-training was 0.0034 ± 0.0026% MNC [0.0014–0.0054% 95% CI] and 0.0030 ± 0.0008% MNC [0.0024–0.0036% 95% CI] (*p* = 0.568, *d* = 0.21). In cells·mL^−1^, this equated to 177 ± 128 [79–275 cells·mL^−1^ 95% CI] and 153 ± 38 cells·mL^−1^ [123–182 cells·mL^−1^ 95% CI] pre- and post-training, respectively (*p* = 0.545, *d* = 0.25).

**Fig. 4:**
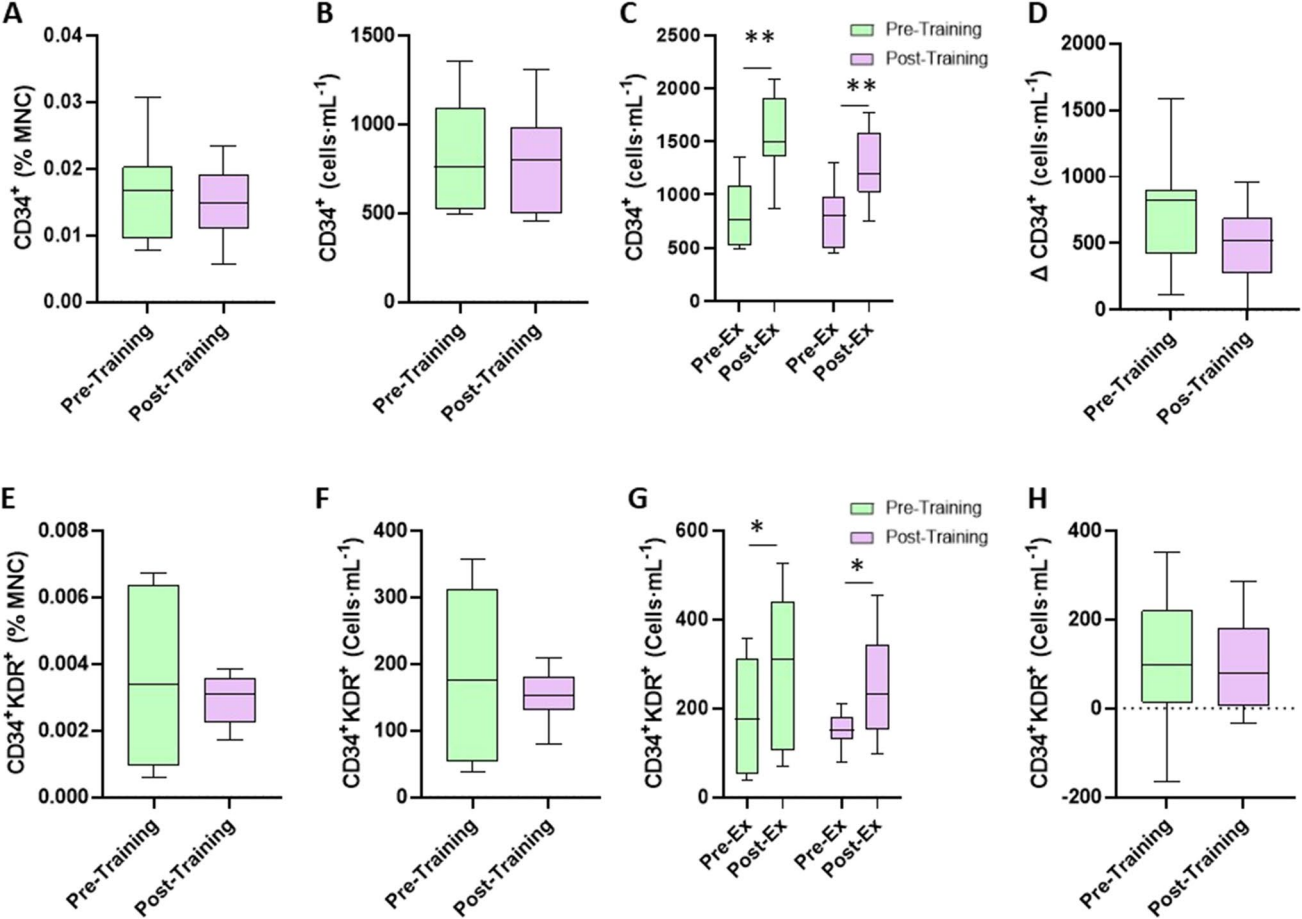
CD34^+^ and CD34^+^KDR^+^ CPC resting and exercise-induced changes in CPC counts in older (*n* = 9) trained adults before and after 8-week SIT intervention. **A**, **B** and **E**, **F** display differences between pre- and post-intervention in CD34^+^ (**A**: % MNC, **B**: cells·mL^−1^), CD34^+^KDR^+^ (**E**: % MNC, **F**: cells·mL^−1^). **C**, **D** and **G**, **H** display exercise-induced changes in CPCs from pre- to post-SIT intervention (**C**: CD34^+^ changes from pre- to post-exercise; **D**: Δ change in CD34^+^ CPCs; **G**: CD34^+^KDR^+^ changes from pre- to post-exercise; **H**: Δ change in CD34^+^KDR.^+^ CPCs). *p < 0.05, **p < 0.005

In terms of graded exercise test to exhaustion-induced changes in CD34^+^ or CD34^+^KDR^+^ CPCs following SIT, the ANOVA resulted in an exercise (i.e., pre- to post-graded exercise test to exhaustion) × phase (i.e., pre and post) interaction of *p* = 0.233 for CD34^+^ CPCs and *p* = 0.921 for CD34^+^KDR^+^ CPCs. The graded exercise test to exhaustion post-SIT resulted in CD34^+^ CPCs of 765 ± 299 [535–995 95% CI] cells·mL^−1^ and 1266 ± 337 [1006–1525 95% CI] cells·mL^−1^ (pre- to post-exercise; *p* < 0.001, *d* = 1.57) and CD34^+^KDR^+^ CPCs of 153 ± 38 [123–182 95% CI] cells·mL^−1^ and 249 ± 121 [156–342 95% CI] cells·mL^−1^ (pre- to post-exercise; *p* = 0.035, *d* = 1.07), and the mean response was not different to that of pre-intervention; however, there was a smaller spread of data, suggesting a more uniform response (Fig. 4).

## Discussion

The main findings of this study are that aerobically trained older adults display a reduced resting number of CPCs compared to younger trained adults but have a preserved ability to mobilise these cells in response to a graded exercise test to exhaustion. Moreover, 8 weeks of SIT did not increase basal CPC counts in already well-trained older adults.

Previous work has shown that advancing age is associated with lower CPC counts [45, 53] which is apparently unaffected by cardiorespiratory fitness [45]. These cells play an important role in vascular repair via promoting endothelial proliferation by paracrine means [22] or by differentiating into mature endothelial cells at the site of repair [61]. Studies report that such CPCs are associated with the endothelial function [4, 19] and as such promote endothelial integrity and health. The loss of such cells with ageing, therefore, results in reduced endothelial repair and loss of endothelial function, which is itself linked heavily with future cardiovascular risk [11]. The mechanism for such reduction in CPCs with advancing age in humans is unknown but purported to be due to increases in oxidative stress, resulting in impairments in CPC number and/or function [32], and increased CPC susceptibility to apoptotic stimuli [26]. There is no evidence for changes in bone marrow resident progenitor cell count with ageing [41], but a stressor-induced CPC mobilisation from bone marrow may be impaired, with evidence from burn wound model of CPC mobilisation [64] and exercise-induced mobilisation both displaying impaired mobilisation in older populations [45]. However, the present investigation observed a preserved exercise mobilisation of CPCs in older adults. There are several key differences which are likely to explain divergent findings. Firstly, participants in this study were a very physically active, trained group of older adults, evidenced by their $\dot{V}$ O_2max_ of 37.4 ml·kg·min^−1^. Previous work by Ross and colleagues 45 which demonstrated impaired mobilisation following exercise in older adults included participants who were not highly physically active, and therefore, the observed reduction in exercise-induced CPC mobilisation may not be a result of age per se, but a result of inactive ageing. Thus, high levels of physical activity throughout the lifespan may be required to preserve this process. In addition to divergent participant characteristics, the exercise stimuli in the work of Ross et al. [45] study was a submaximal cycling protocol at 70% $\dot{V}$ O_2max_, whereas the stressor in the present investigation was a maximal graded exercise test to exhaustion, and CPC mobilisation is intensity dependent [28].

Exercise training has shown promise to promote CPC number and function in both healthy [39, 57] and diseased states [2, 6, 9, 46, 47, 50]. However, some studies report no changes in CPC counts after a short-term training programme [30, 59], likely due to high intra- and inter-group variation associated with quantifying rare cells by flow cytometry. In the present study, we aimed to investigate whether a short-term novel, time-efficient SIT programme could improve the age-related reduction in resting CPC count, and therefore, the trained older adults underwent an 8-week SIT intervention (3 × 20 s ‘all-out’ sprints, 2 × a week). Although SIT has not been well-researched in older adults, HIIT in older adults has been observed to improve cardiorespiratory fitness [25, 51], muscle power [17, 18, 48], and is facilitative in improving body composition [18]. Whilst SIT in younger demographics has been observed to improve both aerobic [49, 60] and anaerobic [24], [31] fitness with a considerable variety of approaches pertaining to interval duration, repetition and training frequency being evidenced as efficacious, whilst remaining easy to administer, i.e. no power or heart monitors required. We observed no change in either resting or exercise-induced changes in CPCs in response to the SIT intervention. We propose that the highly trained status of the older adults was responsible for this, in that the ceiling effect was likely evident in this well-trained group. As such, whether SIT would be beneficial in a sedentary older adult group warrants investigation.

### Limitations

There are several limitations of the current study, which we accept. Firstly, the addition of a sedentary older group and a sedentary younger group to assess the influence of sedentary ageing vs active ageing vs sedentary youth vs active youth more comprehensively would have been beneficial. This would permit us to determine whether the effect of SIT was greater in a physically inactive group, as our older trained group had a CPCs mobilisation capability similar to a young, trained group, contrary to our previous work [45]. However, additional recruitment would require greater resource commitment which was outside the scope of the present investigation. Secondly, whole-body metabolism is largely dependent on skeletal muscle mass, as increased skeletal muscle mass increases metabolic load during rest and exercise, if all other factors are equal. Moreover, as muscle mass ageing and gender have meaningful effects on the physiological stimulus that can be achieved by exercise, mostly attributable to differences in muscle mass between old vs young and male vs female participants [23]. It is likely that older female participants in this study were less muscular than their younger male counterparts, resulting in a relative dampening of the relationship between exercise intensity and metabolic stress [35]. Admittedly, this increased risk of bias in the study results and, consequently, any conclusions derived. Thirdly, we did not assess CPC paracrine function, which improves post-training [27], and therefore, this may be an avenue for future research. Finally, and importantly, this study was not powered to detect changes in CPCs and was a secondary analysis of an investigation with the primary outcome as muscle power. We believe this justifies our statistical approach of avoidance of dichotomous ‘significance’ or otherwise labelling based on an alpha level inappropriate for this dataset. An a posteriori power calculation testing for differences between the young and old group at baseline using CD34 + CPCs (in cells·mL^−1^) as the outcome variable, an alpha of 0.05, a one-sided test, and a sample size of nine resulted in the statistical power of 0.83. Similarly, when using the same information and determining sample size, the required *n* was 10 per group. However, to detect an SIT-induced change in graded exercise test ∆CD34 + (in cells·mL^−1^), a sample size of *n* = 36 would have been required to detect a change at the *p* = 0.05 level, with a statistical power of 0.80 and a one-sided test. Thus, a larger confirmatory study is required to corroborate observations made here.

## Conclusions

Physically trained older adults to display reduced CPC counts but preserved exercise-induced mobilisation of these cells, which could offer vasoprotection. However, an 8-week SIT intervention was unsuccessful at improving resting CPCs and exercise-induced mobilisation of these cells.

## Supplementary Information

Below is the link to the electronic supplementary material.

Supplementary file1 (JPG 113 KB)

Supplementary file2 (PPTX 90 KB)

## Data Availability

The authors contributions in the study are included in the article / supplementary material, further inquiries can be directed to the corresponding authors.

## Abbreviations

ANOVA: Analysis of variance
BLa: Blood lactate
BMI: Body mass index
CPC: Circulating progenitor cell
CV: Cardiovascular
EPC: Endothelial progenitor cell
HIIT: High-intensity interval training
N_2_: Nitrogen
O_2_: Oxygen
PPO: Peak power output
RER: Respiratory exchange ratio
RPE: Rating of perceived exertion
SD: Standard deviation
SIT: Sprint interval training
VO_2_: Oxygen uptake
VO_2peak_: Peak oxygen uptake
