# Supplementary figures and images for: Aerobically trained older adults show impaired resting, but preserved exercise-induced circulating progenitor cell count, which was not improved by sprint interval training
Source: Pflugers Arch. 2023 Feb 14;475(4):465–75. doi: 10.1007/s00424-022-02785-6 (PMC10011317; doi:10.1007/s00424-022-02785-6)

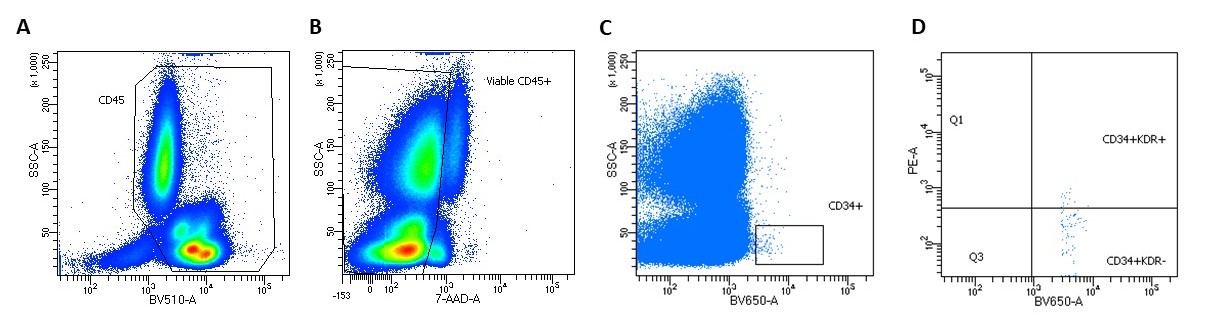

Supplement: Supplementary file 1 — Supplementary file1 (JPG 113 KB) [file 424_2022_2785_MOESM1_ESM.jpg]
