# Supplementary material for: Aerobically trained older adults show impaired resting, but preserved exercise-induced circulating progenitor cell count, which was not improved by sprint interval training
Source: Pflugers Arch. 2023 Feb 14;475(4):465–75. doi: 10.1007/s00424-022-02785-6 (PMC10011317; doi:10.1007/s00424-022-02785-6)
Supplement: Supplementary file 2 — Supplementary file2 (PPTX 90 KB) [file 424_2022_2785_MOESM2_ESM.pptx]

## Slide 1
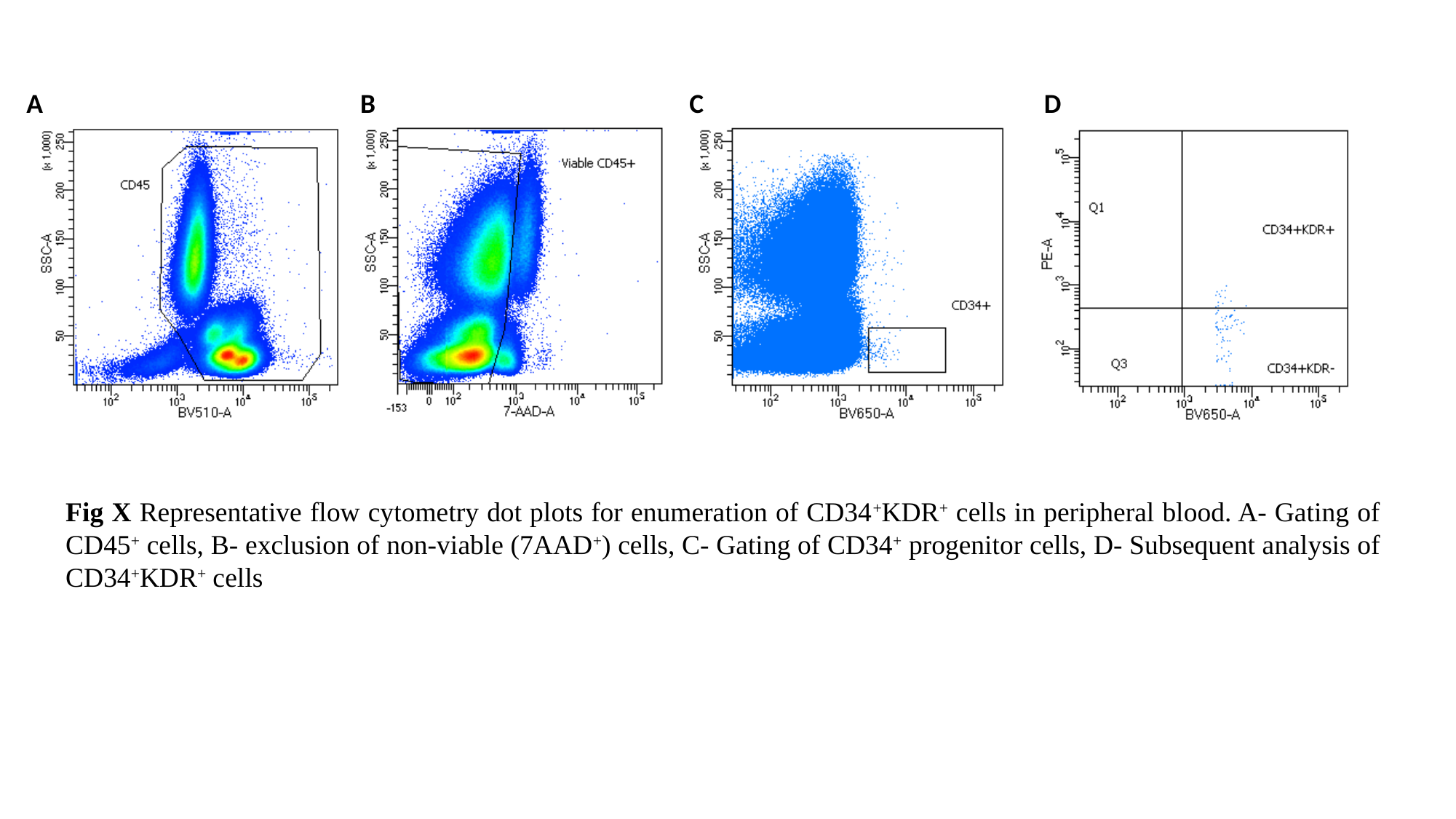

A
B
D
C
Fig X Representative flow cytometry dot plots for enumeration of CD34+KDR+ cells in peripheral blood. A- Gating of CD45+ cells, B- exclusion of non-viable (7AAD+) cells, C- Gating of CD34+ progenitor cells, D- Subsequent analysis of CD34+KDR+ cells
